# Intra-Abdominal Bleeding during Pregnancy, Preterm Delivery, and Placental Polyp in a Long-Term Survivor of Neuroblastoma: A Case Report

**DOI:** 10.1155/2009/564567

**Published:** 2009-12-06

**Authors:** Noriyoshi Watanabe, Junna Tsutsui, Satsuki Kakiuchi, Seung Chik Jwa, Hironori Takahashi, Naomi Kato, Nobuaki Ozawa, Haruhiko Sago, Michihiro Kitagawa

**Affiliations:** Department of Perinatology, National Center of Child Health and Development, 2-10-1 Okura, Setagaya, Tokyo 157-8535, Japan

## Abstract

*Background*. There are few reports of pregnancies in long-term survivors of pelvic neuroblastoma. *Case*. A 30-year-old Japanese woman with a history of pelvic neuroblastoma in her childhood, which was treated with surgical resection, chemotherapy, and radiation. Her pregnancy continued with conservative management, but she delivered a 510 g female infant at 23 weeks of gestation due to sudden onset of labor pain. She also had a placental polyp and developed massive postpartum bleeding. *Conclusion*. Cancer treatment, especially radiation therapy, in childhood may cause adverse outcomes during pregnancy in long-term survivors of neuroblastoma.

## 1. Introduction

Treatment for childhood cancer has become increasingly successful. Approximately 70% of patients with childhood cancer survive for 5 years, and many long-term survivors are now reaching adulthood [1]. Neuroblastoma is a childhood cancer that originates in the adrenal glands in more than 90% of cases. Pelvic neuroblastoma is thought to have a good prognosis, but women who have previously been treated with curative surgery, chemotherapy, or radiation might have long-term effects on their reproductive organs. There are few reports of pregnancies in women who have previously been treated for neuroblastoma. Here, we report a case of a pregnant patient who had been successfully treated for pelvic neuroblastoma.

## 2. Case Report

The patient was a 30-year-old Japanese woman. This was her first pregnancy. She had a pelvic neuroblastoma in the front of her sacrum at 1 month of age. She had undergone surgical resection, and the pathology revealed rosette-forming neuroblastoma. She received adjuvant chemotherapy with vincristine and cyclophosphamide (the James method) postoperatively up to the age of 2 years. She also received pelvic irradiation with a total dose of 18 Gy. By age of 3 years, there was no evidence of disease, and she was considered cured.

**Figure 1:**
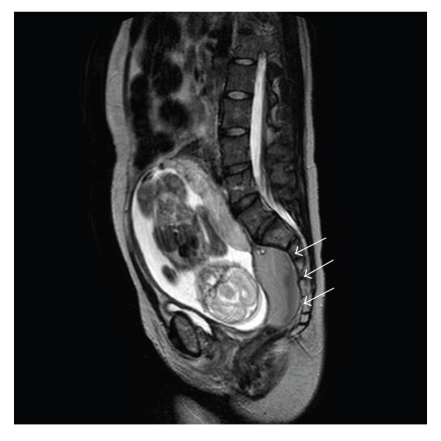
MRI at 21 weeks of gestation. Arrows show location of intra-abdominal bleeding.

She visited out hospital for prenatal care at 6 weeks of gestation. At 15 weeks and 5 days of gestation, she complained of abdominal pain due to frequent uterine contractions and was diagnosed with threatened abortion or incompetent cervix by transvaginal ultrasound. She was admitted at 16 weeks of gestation and ritodrine tocolytic therapy was initiated to control her frequent uterine contractions. At 20 weeks and 2 days of gestation, she developed acute abdominal pain. Ultrasound and magnetic resonance imaging (MRI) revealed intra-abdominal bleeding of unknown etiology (Figure 1). The patient and her husband chose to continue the pregnancy even after we explained to them the risks involved in doing so and after we suggested termination of pregnancy. She received 4 units of red blood cells but did not require any surgical intervention. At 23 weeks of gestation, she prematurely delivered a 510 g female infant due to sudden onset of labor pain. There were no signs of chorioamnionitis. The infant was admitted to the NICU and was discharged in good condition 110 days after birth.

**Figure 2:**
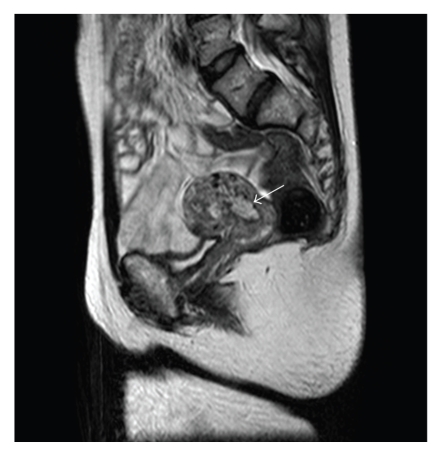
MRI at 47 days after delivery. Arrows point to placental polyp.

The patient presented with massive vaginal bleeding 14 days postpartum. Medical and surgical therapy was performed. Uterine curettage revealed a retained placenta. Despite these treatments, the patient experienced continuous slow vaginal bleeding. However, because her bleeding was light, she waited to see if the bleeding would stop naturally. An MRI was conducted at 46 days postpartum, and the scan revealed a placental polyp (Figure 2). Heavy vaginal bleeding occurred at 49 days postpartum. Uterine artery embolization (UAE) was considered, but the patient was noted to shed the placental polyp spontaneously with full recovery.

## 3. Discussion

Long-term female survivors of childhood pelvic neuroblastoma might develop complications during pregnancy due to the negative effects of prior radiation therapy. Our patient received postoperative chemotherapy with vincristine and cyclophosphamide without apparent effects on her pregnancy. Green et al. [2] reported that prior treatment with specified chemotherapeutic agents does not affect subsequent pregnancy; this finding is also supported by other studies.

Critchley and Wallace [3] reported that radiation therapy could have negative effects on the uterus. Specifically, they reported that radiation doses between 14 and 30 Gy resulted in the impairment of uterine musculature and vasculature and that patient age at the time of irradiation determined the final uterine volume. Our patient delivered at 23 weeks of gestation. Most preterm labor is due to chorioamnionitis; but no evidence of infection was found in our patient. It is possible that the prior radiation to her pelvis resulted in an underdeveloped uterus with subsequent preterm labor and delivery [3, 4]. In this case, because we did not measure her uterine volume, we have no evidence to prove that her uterus was underdeveloped. However, Critchley and Wallace [3] reported that a patient treated with radiation prior to puberty had a small uterus. Therefore, we speculated that our patient's uterus was underdeveloped.

It is possible that the patient's intra-abdominal bleeding might have been a negative consequence of prior radiation. Our patient developed sudden intra-abdominal bleeding at 20 weeks of gestation. The cause is unclear because we did not perform any surgical intervention. The surgery and pelvic irradiation that she underwent in her childhood caused adhesion in her abdomen, and this adhesion may have torn as the uterus extended during pregnancy, resulting in intra-abdominal bleeding at 20 weeks of gestation. 

In this case, pregnancy termination might have to be considered to avoid the associated risk of continuing the pregnancy. We decided to continue her pregnancy for 2 reasons: (i) the patient was determined to give birth to a living infant, and (ii) this pregnancy might have been her last chance to deliver a baby because this atypical complication—intra-abdominal bleeding during her pregnancy—was an effect of pelvic irradiation in her childhood.

Uterine irradiation might predispose a woman to abnormal placentation in a subsequent pregnancy. Norwitz et al. [5] reported a patient with a history of abdominal radiation who experienced placenta percreta and uterine rupture at 17 weeks of gestation. Our patient suffered from placental polyps at almost 2 months postpartum. This finding is also consistent with abnormal placentation and is a likely result of her childhood radiation therapy.

In summary, pregnancy outcomes may be affected in long-term survivors of childhood cancers who have received pelvic radiation. Obtaining a detailed history is essential to manage the pregnancies of such patients. Patients with a history of prior pelvic radiation might experience decreased uterine volume and increased risks of miscarriage, preterm delivery, or abnormal placentation and should receive appropriate high-risk prenatal care.
